# Association of pre-existing depression with all-cause, cancer-related, and noncancer-related mortality among 5-year cancer survivors: a population-based cohort study

**DOI:** 10.1038/s41598-019-54677-y

**Published:** 2019-12-04

**Authors:** Ahryoung Ko, Kyuwoong Kim, Joung Sik Son, Hye Yoon Park, Sang Min Park

**Affiliations:** 1 Department of Family Medicine, Seoul National University Hospital, Seoul, Republic of Korea; 2 Department of Biomedical Sciences, Seoul National University, Seoul, Republic of Korea; 3 Department of Clinical Medical Sciences, Seoul National University, Seoul, Republic of Korea; 4 Department of Psychiatry, Seoul National University Hospital, Seoul, Republic of Korea

**Keywords:** Disease prevention, Outcomes research

## Abstract

Previous studies on the association between mental health and mortality in patients with cancer have reported contradictory results. We conducted a population-based cohort study to determine whether pre-existing depression is associated with all-cause and cause-specific mortality after cancer diagnosis. We included 5-year cancer survivors, identified from the National Health Insurance Scheme Health Screening Cohort between January 1, 2004 and December 31, 2009. Cox proportional hazards models were used to calculate hazard ratios and 95% confidence intervals (CI) to assess the association between pre-existing depression and all-cause, cancer-related, and noncancer-related mortality among 5-year cancer survivors. After adjustment for sociodemographics, lifestyle, and clinical status, the multivariable adjusted hazard ratios (95% CIs) for all-cause, cancer-related, and noncancer-related mortality among 5-year cancer survivors with pre-existing depression were 1.52 (1.13–2.05), 1.17 (0.75–1.81), and 2.07 (1.38–3.10) compared with those without pre-existing depression, respectively. Significant associations between pre-existing depression and mortalities (all-cause and noncancer-related mortality) were only observed among male cancer survivors. Our findings suggest that depression is associated with all-cause mortality after cancer diagnosis and that greater efforts should be focused on the long-term survival of patients with cancer with pre-existing depression, especially in male cancer survivors.

## Introduction

Patients with cancer typically also have other health issues, including cardiovascular diseases (CVD) and mental health problems, as well as risky health behaviors^1^. Whether depression impacts the prognosis of disease is no longer a matter of debate. The mechanisms by which depression affects disease progression and mortality are known to include both behavioral and biological pathways^2–4^. The higher overall mortality among patients with psychiatric disorders may be attributable to a lack of screening or may result from diagnostic delays^5^. This may also result from patients being diagnosed in the more advanced stages of the disease and poor access to treatment, which significantly influences disease outcome^3,6^.

Although previous studies have suggested an association between mental and physical health^7–9^, there is not enough evidence supporting the relationship between pre-existing depression and mortality among cancer survivors^6,10,11^. It has been suggested that, among the factors that increase all-cause mortality, depression is associated with increased cancer- and noncancer-related mortality^5^. Many previous studies have reported that depression is a predictor of the prognosis of cancer course and contributes to increased cancer invasiveness^6,12,13^. In addition to various forms of cancer treatment^14–16^, known risk factors for malignancy, such as tobacco use, physical inactivity, and obesity, which are also risk factors for noncancerous diseases (e.g., CVD, diabetes, and inflammation), increase the risk of chronic health problems other than cancer among cancer survivors^17–19^. Moreover, the number of published studies examining the rates of noncancer-related deaths among adults diagnosed with cancer is limited, even though several previous studies have investigated the long-term outcome of patients with childhood and adolescent cancer^20,21^.

Although several studies have investigated the outcomes of patients with cancer with depression in Western countries^6,22–25^, our study focused on the effect of previously-diagnosed depression on 5-year survival of cancer patients. In this nationally representative, population-based cohort study, we analyzed information regarding diverse health conditions, health behaviors, and biological risk factors. We hypothesized that cancer survivors who had depression before being diagnosed with cancer would have decreased survival rates compared with those without pre-existing depression.

## Results

Between 2004 and 2009, 11,065 (6,023 men [54.4%] and 5,042 women [36.6%]) 5-year CS were identified from the NHIS-HEALS Cohort. Among these 11,065 CS, there were 343 (3.1%) patients with pre-existing depression diagnosed within 2 years prior to the first cancer diagnosis date. More than half of the 5-year CS were aged between 50 and 69 years. Patients with pre-existing depression had more comorbidities (CCI ≥ 2) than patients without pre-existing depression, but there was no significant difference in sociodemographic and lifestyle characteristics. The general characteristics of the 5-year CS are listed in Table 1. Among 343 CS who had pre-existing depression prior to cancer diagnosis, only 57 (16.6%) had medical claims records for depression after cancer diagnosis.

**Table 1:** General characteristics of 5-year cancer survivors of the National Health Insurance Service-Health Screening cohort (2002–2015).

	Total (N = 11,065)	No Pre-existing Depression (N = 10,722)	Pre-existing Depression (N = 343)
Age, years			
40–49	1,899 (17.2)	1,854 (17.3)	45 (13.1)
50–59	3,801 (34.4)	3,697 (34.5)	104 (30.3)
60–69	3,460 (31.3)	3,350 (31.2)	110 (32.1)
≥70	1,905 (17.1)	1,821 (17.0)	84 (24.5)
Sex			
Men	6,023 (54.4)	5,883 (54.9)	140 (40.8)
Women	5,042 (45.6)	4,839 (45.1)	203 (59.2)
Place of Residence			
Metropolitan area	2,067 (18.7)	2,003 (18.7)	64 (18.7)
City/Town	4,946 (44.7)	4,807 (44.8)	139 (40.5)
Country	4,052 (36.6)	3,912 (36.5)	140 (40.8)
Insurance Type			
Employee Insured	7,449 (67.3)	7,229 (67.4)	220 (64.1)
Self-employed	3,467 (31.3)	3,349 (31.2)	118 (34.4)
Medical Aid	149 (1.4)	144 (1.4)	5 (1.5)
Insurance Premium(Quartile)			
1^st^	1,368 (12.4)	1,327 (12.4)	41 (12.0)
2^nd^	2,225 (20.1)	2,150 (20.0)	75 (21.9)
3^rd^	3,270 (29.5)	3,171 (29.6)	99 (28.8)
4^th^	4,202 (38.0)	4,074 (38.0)	128 (37.3)
Presence of Disability			
Yes	116 (1.1)	111 (1.1)	5 (1.5)
No	10,949 (98.9)	10,611 (98.9)	338 (98.5)
Body Mass Index, kg/m^2^			
<25	3,543 (32.1)	3,447 (32.1)	96 (28.0)
≥25	7,522 (67.9)	7275 (67.9)	247 (72.0)
Fasting Serum Glucose, mg/dL			
<126	732 (6.6)	715 (6.8)	17 (4.9)
≥126	10,333 (93.4)	10,007 (93.2)	326 (95.1)
Total Cholesterol, mg/dL			
<240	898 (8.1)	868 (8.1)	30 (8.8)
≥240	10,167 (91.9)	10,722 (96.4)	313 (91.2)
Smoking status			
Non-smoker	8,114 (73.3)	7,837 (73.1)	277 (80.8)
Past smoker	959 (8.7)	933 (8.7)	26 (7.6)
Current smoker	1,654 (15.0)	1,625 (15.0)	29 (8.3)
No Response	338 (3.0)	327 (3.1)	11 (3.3)
Alcohol consumption(times/week)			
1–2	8,749 (79.1)	8,453 (78.9)	296 (86.3)
3–4	1,773 (16.0)	1,740 (16.2)	33 (9.6)
≥5	411 (3.7)	402 (3.7)	9 (2.7)
No Response	132 (1.2)	127 (1.2)	5 (1.4)
Physical activity(times/week)			
1–2	8,620 (77.9)	8,369 (78.1)	251 (73.2)
3–4	1,344 (12.2)	1299 (12.1)	45 (13.1)
≥5	665 (6.0)	637 (5.9)	28 (8.2)
No Response	436 (3.9)	417 (3.9)	19 (5.5)
CCI			
0	3,365 (30.4)	3,300 (30.8)	65 (18.9)
1	4,087 (36.9)	3,984 (37.2)	103 (30.0)
≥2	3,613 (32.7)	3,438 (32.0)	175 (51.1)
Smoking-related cancer	6,414 (58.0)	6,240 (58.2)	174 (50.7)
Obesity-related cancer	5,376 (48.6)	5,237 (48.8)	139 (40.5)
GI cancer	5,109 (46.2)	4,980 (46.4)	129 (37.6)
GU cancer	345 (3.1)	335 (3.1)	10 (2.9)

Data are presented as no (%).

### Association between pre-existing depression and mortality among 5-year CS

During the follow-up period, a total of 1,047 5-year CS died. Among these deaths, 636 (60.7%) were attributable to cancer. The age-adjusted HRs and 95% CIs for all-cause mortality, cancer-related mortality, and noncancer-related mortality among 5-year CS with pre-existing depression were 1.46 (1.10–1.95), 1.06 (0.70–1.63), and 2.07 (1.38–3.10), respectively, compared to those without pre-existing depression. In the multivariate model adjusted for sociodemographic (age, sex, place of residence, insurance type, insurance premium, and presence of disability), lifestyle (cigarette smoking, alcohol consumption, physical activity level), and medical (BMI, FSG, total cholesterol, CCI) characteristics (model 2), pre-existing depression was significantly associated with death by any cause (1.52 [1.13–2.05]) and death by any cause other than cancer (2.07 [1.38–3.10]), but no significant association was found between pre-existing depression and death attributable to cancer (1.17 [0.75–1.81]) among 5-year CS.

In the sex-stratified analysis, a higher percentage of female (4.2%) than male (2.4%) CS had pre-existing depression. However, a significant association between pre-existing depression and mortality was observed only in male CS. The multivariate-adjusted (model 2) HRs and 95% CIs of all-cause mortality for male and female patients were 1.78 (1.25–2.55) and 1.11 (0.94–1.31), respectively. In both male and female patients, pre-existing depression was not significantly associated with cancer-related mortality. The HRs of all-cause and cause-specific mortalities among 5-year CS are presented in Table 2.

**Table 2:** Hazard ratios of all-cause, cancer, and non-cancer death in 5-year cancer survivors.

	Total (N = 11,065)		Men (N = 6,023)		Women (N = 5,042)	
	No Pre-existing Depression (N = 10,722)	Pre-existing Depression (N = 343)	No Pre-existing Depression (N = 5,883)	Pre-existing Depression (N = 140)	No Pre-existing Depression (N = 4,839)	Pre-existing Depression (N = 203)
All-cause Mortality						
No. of Deaths	997	50	710	35	287	15
Person-Years	39,258	1,208	21,150	443	18,108	765
Age-adjusted Model	1	1.46 (1.10–1.95)^***^	1	1.93 (1.38–2.71)^***^	1	1.12 (0.67–1.89)
Model 1^a^	1	1.60 (1.20–2.12)^**^	1	1.93 (1.38–2.72)^***^	1	1.13 (0.67–1.89)
Model 2^b^	1	1.52 (1.13–2.05)^**^	1	1.78 (1.25–2.55)^**^	1	1.11 (0.94–1.31)
Cancer Mortality						
No. of Deaths	614	22	427	14	187	8
Age-adjusted Model	1	1.06 (0.70–1.63)	1	1.31 (0.77–2.23)	1	0.95 (0.49–1.93)
Model 1^a^	1	1.15 (0.75–1.76)	1	1.31 (0.77–2.24)	1	0.95 (0.47–1.92)
Model 2^b^	1	1.17 (0.75–1.81)	1	1.28 (0.74–2.24)	1	0.98 (0.48–2.00)
Non-Cancer Mortality						
No. of Deaths	383	28	283	21	100	7
Age-adjusted Model	1	2.12 (1.44–3.11)^***^	1	2.87 (1.84–4.47)^***^	1	1.47 (0.68–3.17)
Model 1^a^	1	2.34 (1.59–3.44)^***^	1	2.86 (1.84–4.47)^***^	1	1.40 (0.70–3.24)
Model 2^b^	1	2.07 (1.38–3.10)^***^	1	2.47 (1.54–4.00)^***^	1	1.27 (0.57–2.81)

^a^Adjusted for age, sex, place of residence, insurance type, insurance premium, and presence of disability.

^b^Adjusted for age, sex, place of residence, insurance type, insurance premium, and presence of disability, BMI, fasting serum glucose, total cholesterol, cigarette smoking, alcohol consumption, physical activity, and CCI.

Abbreviations: ref, reference category.

NOTE: Statistical significance is noted as *P < 0.05, **P < 0.01, ***P < 0.001.

### Pre-existing depression and all-cause mortality by subgroup

To examine whether there were subgroup effects among the 5-year CS, we stratified the analytic sample into various subgroups of clinical importance. While the overall results were similar to those of the main analysis, the subgroup analysis revealed that CS with pre-existing depression who were older than 60 years, obese (BMI ≥ 25), had elevated FSG (≥126 mg/dL) and cholesterol (≥240 mg/dL) levels, frequently consumed alcohol (≥3 times/week), and had relatively high CCI (≥2) had significantly higher risk of death compared to those without pre-existing depression. The results of the subgroup analysis are listed in Table 3. The association between pre-existing depression and all-cause mortality was assessed across the different types of CS (smoking-related, obesity-related, GI, and GU cancer in Table 4). Additional analysis including the CS with missing variables in the health examination records produced comparable results with those of the main analysis (Supplemental Table 1). The overall results of subgroup analyses were similar to those of the main analysis, although the statistical significance was attenuated.

**Table 3:** Stratified analysis for all-cause mortality in 5 year cancer survivors with pre-existing depression as compared to those without pre-existing depression.

	No Pre-existing Depression	Pre-existing Depression
Age	(ref)	
<60 years	1	1.31 (0.64–2.66)
≥60 years	1	1.61 (1.16–2.23)^**^
Sex	(ref)	
Men	1	1.78 (1.25–2.55)^**^
Women	1	1.11 (0.94–1.31)
Insurance Type^a^	(ref)	
Employee Insured	1	1.62 (1.12–2.33)^*^
Self-employed	1	1.51 (0.95–2.41)
Insurance Premium (Quartile)	(ref)	
1^st^/2^nd^	1	1.41 (0.84–2.38)
3^rd^/4^th^	1	1.61 (1.12–2.31)^**^
Body Mass Index (kg/m^2^	(ref)	
<25	1	0.94 (0.44–2.00)
≥25	1	1.73 (1.26–2.40)^***^
Fasting Serum Glucose (mg/dL)	(ref)	
<126	1	1.12 (0.40–3.11)
≥126	1	1.62 (1.19–2.21)^**^
Cholesterol level (mg/dL)	(ref)	
<240	1	0.38 (0.05–2.84)
≥240	1	1.66 (1.23–2.24)^***^
Alcohol Consumption (times/week)	(ref)	
1–2	1	1.43 (1.04–1.99)^*^
≥3	1	2.15 (1.10–4.24)^*^
Physical Activity (times/week)	(ref)	
1–2	1	1.44 (1.02–2.02)^*^
≥3	1	2.01 (1.09–3.72)^*^
Smoking status	(ref)	
Non-smoker	1	1.64 (1.19–2.29)^**^
Past-smoker	1	1.67 (0.58–4.79)
Current smoker	1	1.11 (0.46–2.71)
Charlson Comorbidity Index	(ref)	
<2	1	1.27 (0.77–2.08)
≥2	1	1.86 (1.29–2.70)^**^

Data above are presented as HR (95%) adjusted for age, sex, place of residence, insurance type, insurance premium, and presence of disability, BMI, fasting serum glucose, total cholesterol, cigarette smoking, alcohol consumption, physical activity, and CCI (except for the variable belonging to the stratified category).

^a^Medical aid category is omitted from the subgroup analysis due to a significantly low number of subjects.

NOTE: Statistical significance is noted as *P < 0.05, **P < 0.01, ***P < 0.001.

**Table 4:** Hazard ratio for all-cause mortality in 5 year cancer survivors with pre-existing depression as compared to those without pre-existing depression categorized by cancer type at inclusion in cohort.

	No Pre-existing Depression	Pre-existing Depression
Cancer Survivors		
Smoking-related	1 (ref)	1.49 (1.05–2.09)^*^
Obesity-related	1 (ref)	1.59 (1.08–2.33)^*^
GI	1 (ref)	1.58 (1.06–2.36)^*^
GU	1 (ref)	2.51 (0.66–9.57)

Data above are presented as HR (95%) adjusted for age, sex, place of residence, insurance type, insurance premium, and presence of disability, BMI, fasting serum glucose, total cholesterol, cigarette smoking, alcohol consumption, physical activity, and CCI.

Abbreviations: GI, gastrointestinal; GU, genitourinary; ref, reference.

NOTE: Statistical significance is noted as *P < 0.05, **P < 0.01, ***P < 0.001.

### Sensitivity analysis

When the time-window of detecting depression prior to cancer diagnosis among 5-year CS was extended to 3 years, the overall association of pre-existing depression prior to cancer diagnosis and mortality remained similar to the main analysis. However, no statistical significance was found for each category of mortality in this analysis. Further adjusting for depression after cancer diagnosis in the multivariable Cox proportional hazards models did not alter the associations found in the main analysis.

## Discussion

This population-based longitudinal study revealed a significant association between pre-existing depression and long-term mortality in 5-year CS. To our knowledge, this study is the first to show that pre-existing depression is associated with an increased risk of mortality, particularly increased noncancer-related mortality, among LCS.

Studies have reported that depression is implicated in morbidity and mortality in patients with cardiovascular disease, diabetes, and cancer^5,8,26,27^; these findings are consistent with those of the current study on CS. Indeed, the risk of all-cause mortality was 46–52% higher among patients with than without pre-existing depression (Table 2).

The higher mortality rate found among patients with depression might be attributable to the mechanisms specific to the existing disease^28^. In cancer, one of the suggested disease-specific mechanisms is stress affecting the incidence of cancer by impacting the repair of damaged DNA^29^ and promoting tumor progression by accelerating tumor cell growth^30^, which subsequently results in poorer cancer outcomes^31^. Our study revealed that 5-year CS with pre-existing depression had higher overall mortality than did those without pre-existing depression; however, the risk of cancer-related mortality was not significantly different. This finding was consistent with those of previous studies reporting that the association between depression and cancer-related mortality decreases when the follow-up period exceeds 5 years^11,23^, suggesting that other intervening factors are more likely to be responsible for the increased long-term mortality.

The present findings further extend the results of previous studies reporting that noncancer-related deaths, rather than cancer-related deaths, are the main factors for increased overall mortality in long-term cancer survival. In our study, although the sample did not suffice to determine noncancer-related death causes, it is reasonable to assume that poorly-controlled patients such as those with a history of depression may have worse medical status, ultimately leading to an elevated risk of noncancer-related mortality among LCS. Noncancer-related mortality and lower disease specificity to cancer have been proposed in psychiatric patients with somatic disease adverse effect on adherence to prescribe a treatment regimen, and unhealthy behaviors^32^ may influence the mortality rate. Moreover, depression reportedly has adverse effects on physiological changes, including on neuroendocrine and immunological processes. Cardiac rhythm disturbances and localized inflammation may be factors associated with physical health deterioration, which increases the risk of morbidity including CVD^8,33,34^, which is a leading non-malignant cause of death among CS. Patients with chronic illness (e.g., hypertension, chronic obstructive pulmonary disease, and diabetes) and depression are more functionally disabled, have poorer health perception, and poorer wellbeing than those with chronic illness alone^35,36^.

The mechanisms by which depression affects mortality are known to include behavioral pathways (treatment adherence and health behaviors) and biological pathways (e.g., the neuroendocrine and neuro-immunological systems and the circadian rhythm)^2^. A review of the literature showed that several studies have examined the survival rate during cancer treatment taking into account pre-existing depression^6,37^. However, to examine the influence of pre-existing depression on long-term mortality, it is necessary to have evidence showing that pre-existing depression can consistently affect the above-mentioned pathways and that depression can have future behavioral and physiological consequences^2,4^.

The results of the subgroup analyses revealed that the mechanism by which depression affects mortality possibly includes biological and behavioral pathways. This study showed that mortality was higher among patients with than without depression within the groups of poor endocrine factors (aging, obesity, cholesterol, or FSG levels) (Table 3). The effect of depression on mortality can be observed more overtly among patients with cancer with impaired regulation of the endocrine system^9,38^. The composition of the body changes with aging and involves increased body fat mass resulting in functional limitations among the elderly^39^. These may reduce motivation to undergo depression treatment, ultimately affecting mortality. Considering the behavioral pathways involved in depression, these findings suggest that depression can lower treatment adherence or adversely affect diet control, which can indirectly exacerbate disease progress in patients with cancer^40^. Moreover, depression had a greater effect on mortality, especially noncancer-related mortality, among men than among women (Tables 2 and 3). It is considered that men require more time to acknowledge emotional changes, and thus psychiatric treatment for their emotional problems is also delayed^41,42^, which may lead to suboptimal treatment adherence. The present study considered health behaviors, such as alcohol consumption, smoking, and physical activity, among the underlying behavioral pathways and showed that high mortality was observed among patients with depression only when frequent alcohol consumption was involved. Furthermore, there was no association between decreased survival rate and unhealthy behaviors when the smoking pattern and physical activity level were considered. Since the numbers of non-smokers and individuals with regular physical activity (more than 3 times per week) were high in our study sample, it is possible that the risk for mortality remained statistically significant in the stratified analyses. There is uncertainty regarding the underlying mechanisms related to both biological changes and health behaviors involved in depression; thus, our results form an important basis for explaining the mechanisms underlying depression and mortality increase.

Even though this study is limited in that the clinical stages were not reflected and individual analysis for each type of cancer was impossible because of the small number of cases, we conducted stratified analyses based on several cancer categories. A similar trend was observed for patients with gastrointestinal, smoking-related, and obesity-related cancer, although the correlation coefficients were slightly lower than those obtained from the main analysis (Table 4). Few studies have compared the effect of depression on the risk of death among patients with and without depression based on cancer site. Our study population was limited to patients with cancer who survived for 5 or more years, and we demonstrated that the effect of pre-existing depression on long-term cancer mortality was preserved among the subgroups of cancer.

Some limitations of our study need to be considered when interpreting the results. Because the stage and type of cancer treatment were not available, their effects on mortality could not be determined. The present study showed that there was no difference in cancer mortality between the groups with and without depression. This may be attributed to the fact that factors such as clinical stage and curative treatment, which have a much stronger effect on survival rate were not corrected for or to the effects of depression having been offset by these factors.

Furthermore, the use of psychiatric medications, marital status, education level, and employment status were not recorded for the cohort and could not be included in our models. However, we adjusted for the CCI in our analysis to account for comorbidities associated with the cancer outcome. In addition, although a recent meta-analysis reported that a diagnosis of depression is slightly more strongly associated with mortality than are depressive symptoms alone^24^, this measure may not be sensitive enough to identify patients with depression because physicians frequently overlook this condition and medical insurance coverage for mental health benefits is limited. In addition, our data did not include or assess the stress levels of CS with pre-existing depression. The present study, which was conducted with consideration to various confounders to show the effects of depression on decreased survival rate among patients with cancer, should be complemented by other studies considering these factors to confirm whether the results are consistent.

While previous studies investigated the association between depression and cancer in the general population, focused on only specific type of cancer survivor, based their study on cancer patients with coexisting depression, our analytic sample was limited to 5-year CS with all cancer sites and examined the association of pre-existing depression and cause-specific mortality using a routinely collected medical claims data^22–25^. Therefore, our findings on the association of pre-existing depression and all-cause mortality provides additional evidence that treatment of pre-existing depression is of an important clinical intervention for survival of cancer patients. Our study suggests that pre-existing depression among patients with cancer should be assessed in addition to the known risk factors associated with long-term mortality. To prove a causal effect of depression on survival, there is need for longitudinal studies combining sufficient instrumental properties to measure depression and its presumed pathophysiological mechanisms, followed by adequately powered, randomized trials targeting the implicated mechanisms.

## Methods

### Data source

The National Health Insurance Service (NHIS) Health Screening (HEALS) Cohort is a population-based cohort that was established by the NHIS in 2002 for research purposes. Under a single-insurer system in the Republic of Korea, the NHIS collects data on sociodemographics (age, sex, average insurance premium, residential area, presence of disability), clinical information (comorbidities, number of outpatient visits, and hospitalization records), and the results from a national health screening program. The NHIS HEALS Cohort includes 514,866 participants (approximately 10% of the Korean population who were older than 40 years and were eligible for national health screening in January 2002) who were followed for 13 years, until December 2015.

### Study population

Our study included 5-year CS identified from the NHIS HEALS Cohort between January 1, 2004 and December 31, 2009. CS were identified from the NHIS claims records for hospital admission for cancer using the International Classification of Disease, 10th revision (ICD-10) and those who were alive 5 years after the first cancer diagnosis date were defined as 5-year CS. Inclusion criteria for patients with cancer (claims codes for hospital admission for cancer) in the NHIS claims data were adopted from the previous study comparing the cancer incidence rates between the NHIS and the Korea National Cancer Incidence Database^43^. Among 13,145 5-year CS, 2,080 patients with missing data regarding the results of the national health screening were excluded. Therefore, our study included 11,065 5-year CS. This study was approved by the Institutional Review Board of Seoul National University Hospital (IRB number: 1604-100-756). The requirement for informed consent was waived due to the fact that the NHIS-HEALS is anonymized in adherence to strict confidentiality guidelines.

### Identification of pre-existing depression

Pre-existing depression among 5-year CS was defined as any first-ever psychiatric admission or outpatient visit with at least one of the health claims being for depression (ICD-10: F32 [Major Depressive Disorder] or F33 [Recurrent Depressive Disorder]) within 2 years before the first diagnosis of cancer. The relevant records were retrieved from the NHIS health claims data of the patients.

### Outcomes

The primary outcomes of this study were all-cause mortality, cancer-specific mortality, and noncancer-related mortality among 5-year CS. The causes of death (recorded with ICD-10) were obtained from the death records of Statistics Korea and linked to the relevant data of the NHIS-HEALS Cohort. In our study sample, data on time of death by any cause, cancer-related (ICD-10: C00–C97) death, and death by any cause other than cancer were obtained from the linked database.

### Covariates

Sociodemographic information (age, place of residence, insurance type, insurance premium, and presence of disability) of 5-year CS in our study was collected from the NHIS-HEALS Cohort data for insurance eligibility at the year of cancer diagnosis. Data on lifestyle (smoking status, alcohol consumption, and physical activity level) and medical characteristics (body mass index [BMI], fasting serum glucose [FSG], total cholesterol, and Charlson Comorbidity Index [CCI]) of the CS were collected from the national health screening and health claims records prior to the first cancer diagnosis date. CS were further classified to the following categories. Smoking-related cancer included head and neck (C00-C14), esophagus (C15), stomach (C16), colorectum (C18-C20), liver (C22), pancreas (C25), larynx (C32), trachea (C33), lung (C34), bladder (C67), kidney, kidney pelvis, or ureter (C64-C66, C68); and acute myeloid lymphoma (C92). Obesity-related cancer included endometrial (C54), esophageal adenocarcinoma (C15), stomach (C16), liver (C22), kidney (C64), multiple myeloma (C90), meningioma (C32), pancreas (C25), colorectal cancer (C18-20), gallbladder (C23), breast (C50), and ovarian (C56). Gastrointestinal cancer included the ICD 10 codes C15–C26, and genitourinary cancer included codes C60–C68.

### Statistical analysis

For the 5-year CS, follow up began within 5 years after the date of first cancer diagnosis and lasted until the date of death by any cause, cancer-related death, death by any cause other than cancer (noncancer), or December 31, 2015. We used Cox proportional hazards regression to estimate the hazard ratios (HRs) and 95% confidence intervals (95% CIs) for all-cause mortality, cancer-related mortality, and noncancer-related mortality among 5-year CS with and without pre-existing depression. Proportionality assumption of the model was tested graphically with log-log plot and also with Schoenfeld residuals for testing the independence between residuals and time. First, we developed an age-adjusted model and conducted sex-stratified analysis. For the multivariate analyses, we developed multivariate model 1, adjusting for sociodemographic variables (age, sex, place of residence, insurance type, insurance premium, and presence of disability), and model 2, adjusting for lifestyle (cigarette smoking, alcohol consumption, physical activity level) and medical characteristics (BMI, FSG, total cholesterol, and CCI) in addition to the variables included in model 1. Moreover, we conducted subgroup analyses stratified by age (<60 years, ≥60 years), sex (men, women), insurance type (employee insured, self-employed), insurance premium (1^st^, 2^nd^, 3^rd^, and 4^th^ quartile), BMI (<25, ≥25 kg/m2), FSG (<126, ≥126 mg/dL), cholesterol level (<240, ≥240 mg/dL), alcohol consumption (1–2, ≥3 times/week), physical activity level (1–2, ≥3 times/week), smoking status (nonsmoker, past smoker, current smoker), and CCI (<2, ≥2). We also stratified the study population by type of cancer at enrollment (smoking-related, obesity-related, gastrointestinal, or genitourinary cancer). In addition, we included those with missing variables in the health examination records and conducted the same analyses only adjusting for sociodemographic variables and comorbidities. To investigate the potential bias from the time-window of depression detection prior to cancer diagnosis and depression after cancer diagnosis on the association of pre-existing depression and mortality among 5 year CS, we extended the period to 3 years prior to cancer diagnosis and adjusted depression after cancer diagnosis as a binary variable in the sensitivity analysis. All p-values were two-sided and a p-value < 0.05 was considered statistically significant. We used SAS version 9.3 (SAS Institute Inc, Cary, NC, USA) for data analyses.

## Supplementary information

Supplemental Table 1
