# Supplementary material for: Association of pre-existing depression with all-cause, cancer-related, and noncancer-related mortality among 5-year cancer survivors: a population-based cohort study
Source: Sci Rep. 2019 Dec 4;9:18334. doi: 10.1038/s41598-019-54677-y (PMC6892796; doi:10.1038/s41598-019-54677-y)
Supplement: Supplementary file 1 — Supplemental Table 1 [file 41598_2019_54677_MOESM1_ESM.docx]

**Supplementary information**

**Title**

**Association of pre-existing depression with all-cause, cancer-related, and noncancer-related mortality among 5-year cancer survivors: a population-based cohort study**

Ahryoung Ko, Kyuwoong Kim, Joung Sik Son, Hye Yoon Park, Sang Min Park

Supplemental Table 1. Hazard ratios of all-cause, cancer, and non-cancer death in 5-year cancer survivors (including those with missing variables of health examination records) in the NHIS Screening Cohort.

|  | Total (N=13,145) | | Men (N=7,070) | | Women (N=6,075) | |
| --- | --- | --- | --- | --- | --- | --- |
|  | No Pre-existing Depression  (N=12,746) | Pre-existing  Depression  (N=399) | No Pre-existing Depression  (N=6,906) | Pre-existing Depression  (N=164) | No Pre-existing Depression  (N=5,840) | Pre-existing Depression  (N=235) |
| All-cause Mortality |  |  |  |  |  |  |
| No. of Deaths | 1,209 | 58 | 855 | 40 | 354 | 18 |
| Person-Years | 45,613 | 1,370 | 24,233 | 507 | 21,379 | 863 |
| Age-adjusted Model | 1 (ref) | 1.48 (1.14-1.92)^**^ | 1 (ref) | 1.85 (1.35-2.55)^***^ | 1 (ref) | 1.21 (0.76-1.95) |
| Multivariate Model^a^ | 1 (ref) | 1.39 (1.07-1.81)^**^ | 1 (ref) | 1.78 (1.29-2.44)^***^ | 1 (ref) | 1.10 (0.68-1.78) |
| Cancer Mortality |  |  |  |  |  |  |
| No. of Deaths | 737 | 29 | 516 | 18 | 221 | 11 |
| Age-adjusted Model | 1 (ref) | 1.23 (0.85-1.78) | 1 (ref) | 1.41 (0.88-2.25) | 1 (ref) | 1.20 (0.65-2.19) |
| Multivariate Model^a^ | 1 (ref) | 1.20 (0.83-1.74) | 1 (ref) | 1.39 (0.87-2.23) | 1 (ref) | 1.14 (0.62-2.11) |
| Non-Cancer Mortality |  |  |  |  |  |  |
| No. of Deaths | 472 | 29 | 339 | 22 | 133 | 7 |
| Age-adjusted Model | 1 (ref) | 1.90 (1.30-2.76)^**^ | 1 (ref) | 2.53 (1.65-3.90)^***^ | 1 (ref) | 1.28 (0.60-2.75) |
| Multivariate Model^a^ | 1 (ref) | 1.67 (1.15-2.45)^**^ | 1 (ref) | 2.32 (1.51-3.59)^***^ | 1 (ref) | 1.03 (0.48-2.23) |

^a^Adjusted for age, sex, place of residence, insurance type, insurance premium, and presence of disability and CCI

Abbreviations: ref, reference category

NOTE: Statistical significance is noted as *P<0.05, **P<0.01, ***P<0.001
